# Prediction for electronic, vibrational and thermoelectric properties of chalcopyrite AgX(X=In,Ga)Te_2_: PBE + U approach

**DOI:** 10.1098/rsos.170750

**Published:** 2017-10-04

**Authors:** Jianhui Yang, Qiang Fan, Xinlu Cheng

**Affiliations:** 1 School of Physics and Electronic Engineering, Leshan Normal University, Leshan 614004, People's Republic of China; 2 Institute of Atomic and Molecular Physics, Sichuan University, Chengdu 610065, People's Republic of China

**Keywords:** first principles, lattice dynamics, thermoelectric properties, Ag-based chalcopyrite materials

## Abstract

The electronic, vibrational and thermoelectric transport characteristics of AgInTe_2_ and AgGaTe_2_ with chalcopyrite structure have been investigated. The electronic structures are calculated using the density-functional theory within the generalized gradient approximation (GGA) of Perdew–Burke–Ernzerhof functional considering the Hubbard-U exchange correlation. The band-gaps of AgInTe_2_ and AgGaTe_2_ are much larger than previous standard GGA functional results and agree well with the existing experimental data. The effective mass of the hole and the shape of density of states near the edge of the valence band indicate AgInTe_2_ and AgGaTe_2_ are considerable p-type thermoelectric materials. An analysis of lattice dynamics shows the low thermal conductivities of AgInTe_2_ and AgGaTe_2_. The thermoelectric transport properties' dependence on carrier concentration for p-type AgInTe_2_ and AgGaTe_2_ in a wide range of temperatures has been studied in detail. The results show that p-type AgInTe_2_ and AgGaTe_2_ at 800 K can achieve the merit values of 0.91 and 1.38 at about 2.12 × 10^20^ cm^−3^ and 1.97 × 10^20^ cm^−3^ carrier concentrations, respectively. This indicates p-type AgGaTe_2_ is a potential thermoelectric material at high temperature.

## 1. Introduction

Thermoelectric material, which can be used in thermoelectric devices to convert waste heat into electricity without any mechanical component, has garnered great attention in recent years given the challenges brought about by the global energy crisis. The conversion efficiency between heat and electricity of thermoelectric material is represented by a dimensionless thermoelectric figure of merit (ZT) [1]. The ZT value is determined by the Seebeck coefficient (*S*), electrical conductivity (*σ*), absolute temperature (*T*) and thermal conductivity (*κ*), and defined as: ZT = *S*^2^*σT*/*κ*. The thermal conductivity includes contributions by two parts, the electron and the phonon. The ZT value can be improved by increasing the power factor (PF = *S*^2^*σ*) or decreasing thermal conductivity. The PF can be improved by using ‘band engineering’ to increase the effective mass and carrier concentration optimization [2–6]. The lattice thermal conductivity can be decreased by using some strategies to increase the scattering of phonon waves such as the introduction of lattice imperfection [7] or nanoscale and mesoscale crystal structures [8–12]. Because of the dependent interrelation of the Seebeck coefficient, electrical conductivity via carrier concentration and effective mass poses a great challenge to regulate the thermal and electrical properties of thermoelectric material.

Ag-based ternary chalcopyrite semi-conductive materials have been widely studied regarding their potential applications such as optoelectronic, nonlinear optical devices and photovoltaic solar cells, due to their suitable direct band-gap and large absorption coefficients [13–15]. The measured values of thermal conductivity for AgGaTe_2_ and AgInTe_2_ at room temperature are 1.94 and 2.05 W m^−1^ K^−1^, respectively [16]. Their low thermal conductivities make AgGaTe_2_ and AgInTe_2_ promising thermoelectric materials. The thermal properties of AgGaTe_2_ including Debye temperature entropy and heat capacity under different pressure and temperature have been calculated by Sharma *et al*. [17]. The thermoelectric transport characteristic depends not only on temperature but also on carrier concentration. The thermoelectric transport characteristic is needed to provide the reference doping carrier concentration for further experimental research and promote the application of thermoelectric material. Peng *et al*. [18] and Parker *et al*. [19] have found that AgGaTe_2_ is a promising thermoelectric material in p-type doping with a carrier concentration of the order of 10^19^–10^20^ cm^−3^. However, they obtained the electrical conductivity with respect to relaxation time (*σ*/*τ*) using the Boltzmann transport theory based on the electronic eigenvalues from generalized gradient approximation (GGA), and have not obtained the ZT values of AgGaTe_2_.

Based on the self-consistent electronic eigenvalues calculated from first principles, the thermoelectric transport power functions, *S* as well as *σ*/*τ*, can be straightforwardly obtained from Boltzmann's transport theory without any adjustable parameter. As is well known, the standard GGA often significantly underestimates band-gap. In the case of AgGaTe_2_, The calculated band-gap was only 0.075 eV within standard GGA [18], which is much smaller than the experimental value (1.2 eV) [20]. On the other hand, GGA cannot successfully describe the silver 4d-electron states for the strong correlation [21–23]. So, to accurately and comprehensively analyse the thermoelectric transport properties of AgGaTe_2_ and AgInTe_2_ from accurate electronic eigenvalues, we adopt the PBE + U approach to overcome these issues. The so-called PBE + U approach means including an intra-site Coulomb repulsion U-term by considering the strong exchange correlation to GGA of Perdew–Burke–Ernzerhof (PBE) functional.

In addition, previous research has not taken into account variations in thermoelectric performance in different crystallographic directions for AgGaTe_2_ and AgInTe_2_. This compels us to calculate in detail the thermoelectric properties depending on the carrier concentration and temperature of AgGaTe_2_ and AgInTe_2_ in different crystallographic directions using PBE + U approach.

This paper is organized as follows. The details of our calculational method and computational process are presented in §2, and the results and a discussion on the electronic structures, phonon dispersion relations and thermoelectric transport properties are presented in §3. Section 4 is the conclusion.

## 2. Method of calculation

The structure optimization and electronic properties are performed in the form of the density-functional theory (DFT) using the Vienna Ab-initio Simulation Package (VASP) code [24,25] within GGA-PBE functional form [26]. 4d^10^5s^1^, 4s^2^4p^1^, 5s^2^5p^1^ and 5s^2^5p^4^ are chosen as valence electrons for Ag, Ga, In and Te, respectively. To treat the strong correlation of Ag-4d electrons, the Hubbard-U correction in Dudarev's approach [27] is adopted. In Dudarev's approach, the Coulomb and exchange interactions are specified by the *U*_eff_ parameter. The value of *U*_eff_ = 4.5 eV is used for Ag-4d electrons from previous work on silver oxide [28].

A 7 × 7 × 7 k-mesh in primitive cell, centred at *Γ*, is chosen for integrations in the first Brillouin zone for the total energy and density of states (DOS). The cut-off energy of the electron wave function set is 400 eV. The total energy convergence threshold on each atom is lower than 1 × 10^−6^ eV. The maximal force on each ion is less than 5 meV Å^−1^ for relaxation both atomic positions and cell parameters. The phonon transports have been investigated in the form of harmonic approximation and supercell approach. We perform 2 × 2 × 2 supercell including 128 atoms and 0.01 Å atomic displacement distance to calculate the second-order force constants by using VASP code. Based the calculated force constants, the phonon dispersion relations are computed along high-symmetry points in the first Brillouin zone by Phonopy code [29]. The BoltzTraP code [30] has been employed to obtain the thermoelectric transport functions such as Seebeck coefficients (*S*) and electronic conduction with respect to scattering time (*σ*/*τ*) using first-principles data. This method is based on the Boltzmann transport theory in the form of constant scattering time approximation (CSTA). To help obtain correct transport properties, much denser *k*-mesh (19 × 19 × 19 in primitive cell) is used to ensure the accuracy of self-consistent energy values. The relaxation time has been adjusted from existing experimental data to obtain the electronic conduction (*σ*).

## 3. Results and discussion

**Figure 1.**
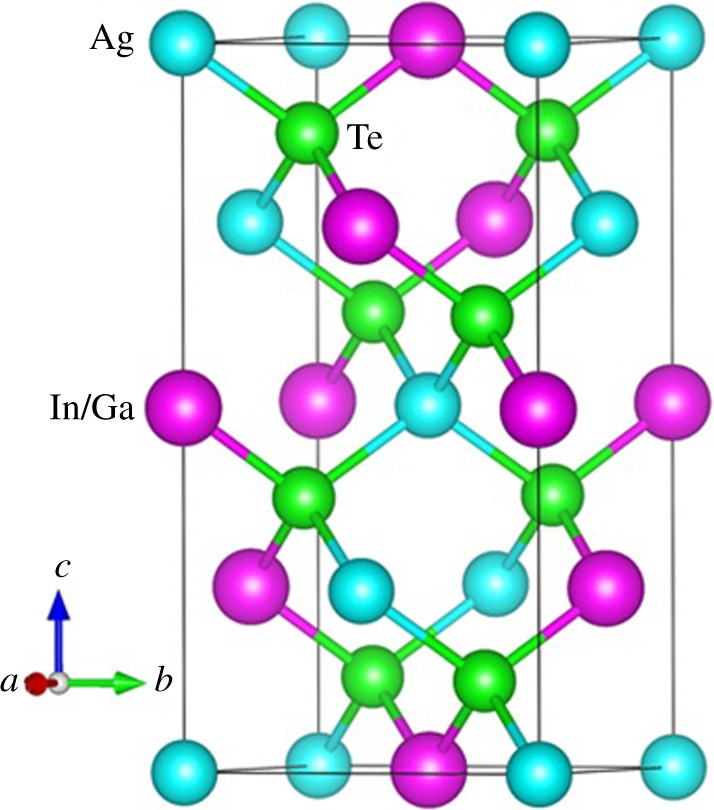
Crystal structure of AgX(In,Ga)Te_2_.

The ternary chalcopyrite structure compound AgX(In,Ga)Te_2_ is crystallized in the tetragonal phase with space group $I\bar{4}2\text{d}$ (no. 122). The crystal structure of AgX(In,Ga)Te_2_ is shown in figure 1 using VESTA software [31]. There are four formula unit atoms in each unit cell. Each Ag or In/Ga atom connects with four Te atoms to form a diamond-like structure.

The optimized structural constants for AgInTe_2_ are *a* = *b* = 6.56 Å and *c* = 12.65 Å, which are in substantial agreement with previously reported experimental results of *a* = *b* = 6.44 Å, *c* = 12.64 Å [32] and theoretical values [33]. The optimized lattice constants (*a* = *b* = 6.39 Å, *c* = 11.82 Å) for AgGaTe_2_ are in excellent agreement with experimental values within a 1% mismatch [18].

The Seebeck coefficient is directly proportional to the effective mass of carriers and slope of the DOS near the Fermi level [34,35]. The chalcopyrite structure semiconductors have a direct band-gap at the *Γ* point [36–39]. The shapes of the calculated band structures are similar to the previous DFT results and expected band-gap [40] and thus no more considered in this paper. In this paper, we give insight into the effective masses of holes and electrons at the valence band maximum (VBM) and conduction band minimum (CBM). The effective masses of holes and electrons at the *Γ* point along different crystallographic directions have been calculated using: $(1\text{/}m^{*})=(1\text{/}\hbar^{2})(\partial^{2}E\text{/}\partial k^{2}),$ where ℏ,E and k are the reduced Planck constant, energy eigenvalue and wavevector, respectively. For example, the effective masses of holes and electrons for AgX(In,Ga)Te_2_ along the *c*-direction can be obtained from the VBM and CBM of band structure along *Z*(0.5, 0.5, −0.5)–*Γ*(0, 0, 0)–*Z*(0.5, 0.5, −0.5) path. The effective masses of holes and electrons for AgX(In,Ga)Te_2_ along the *a-* and *c*-directions are listed in table 1. The data from table 1 indicate the effective masses of holes and electrons for the chalcopyrite structure compound AgX(In,Ga)Te_2_ are anisotropic. We find the effective masses of holes and electrons along the *c*-direction are the same, while they are different along the *a*-direction. In addition, we noted that the effective mass of holes is larger than the effective mass of electrons along the *a*-direction. Hence, a large Seebeck coefficient for p-type AgX(In,Ga)Te_2_ along the *a*-direction is anticipated.

**Table 1.** Effective masses (in unit of *m*_e_) of holes and electrons for AgX(In,Ga)Te_2_ along *a-* and *c*-directions.

	AgInTe_2_		AgGaTe_2_	
	*a*	*c*	*a*	*c*
hole	0.59	0.04	0.57	0.05
electron	0.05	0.04	0.08	0.05

**Figure 2.**
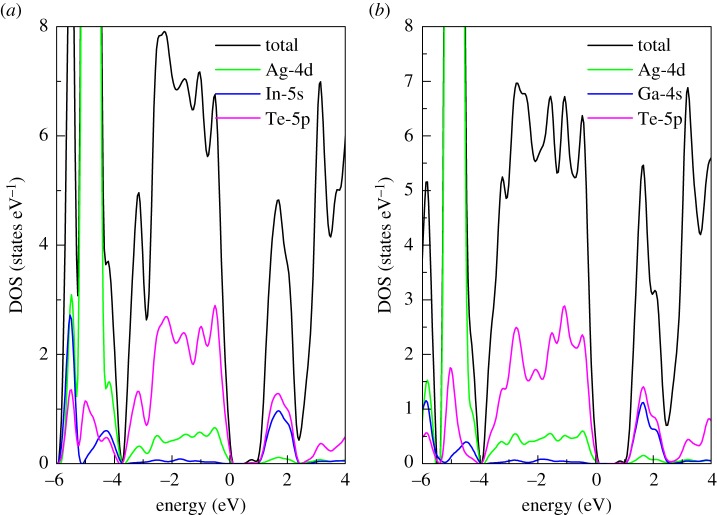
Calculated partial and total DOS for (*a*) AgInTe_2_ and (*b*) AgGaTe_2_ (Fermi level is set at 0 eV).

The partial and total DOS for AgX(In,Ga)Te_2_ are shown in figure 2. We can see that the calculated band-gap energies using PBE + U approach for AgInTe_2_ and AgGaTe_2_ are 0.99 eV and 1.04 eV, respectively. Our calculated results agree well with previous experimental data [14,20], which inspires confidence in the accuracy of our calculation. Moreover, the Ag-4d and Te-5p states are the main contributors to VBM. The CBM is chiefly determined by the mixture between Te-5p and In-5s or Ga-4s states. Meanwhile, as can be clearly seen from figure 2, DOS near the edge of the valence band is slightly steeper than that near the edge of the conduction band. The steep DOS leads to large Seebeck coefficient [41], which indicates AgX(In,Ga)Te_2_ may be possible p-type thermoelectric materials.

**Figure 3.**
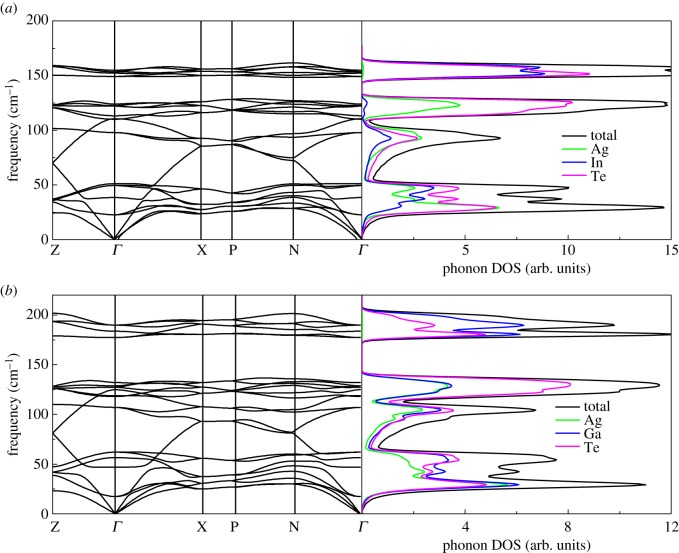
Calculated phonon dispersion curves and projected phonon DOS for (*a*) AgInTe_2_ and (*b*) AgGaTe_2_.

Now, we turn to the lattice dynamical properties of the chalcopyrite structure compound AgX(In,Ga)Te_2_. The vibrational spectrum curves together with the corresponding projected phonon DOS for AgX(In,Ga)Te_2_ are plotted in figure 3. As there are eight atoms in a primitive cell, the phonon dispersion curves involve 24 phonon modes. The positive phonon frequencies suggest AgInTe_2_ and AgGaTe_2_ are dynamically stable. The phonon dispersion relations for AgInTe_2_ and AgGaTe_2_ are similar to each other. Both phonon dispersion relations of the two compounds have frequency gaps and consist of two groups of bands. The high-frequency optical modes (up to 145 cm^−1^ and 173 cm^−1^, respectively, for AgInTe_2_ and AgGaTe_2_) are determined by the contributions of In/Ga and Te atoms. The low frequencies up to the frequency gap are mixed by the Ag, Te and In/Ga atoms. Compared with flat optic phonon modes, highly dispersive acoustic phonon modes have larger group velocities. Moreover, there are some low-frequency (below 50 cm^−1^) optic phonon modes mixing with acoustic phonon modes. The mixture of optic and acoustic phonon modes promotes the reduction of thermal conductivity. We find that the phonon DOS of AgGaTe_2_ is a little wider than that of AgInTe_2_ in the low-frequency range of up to 50 cm^−1^ and the Ga atom contributes to moving towards a lower frequency because of the lighter mass. In addition, the minimum frequency of the optic mode for AgGaTe_2_ is 17.8 cm^−1^, while the frequency of the corresponding mode for AgInTe_2_ is 22.7 cm^−1^, which suggests that AgGaTe_2_ has smaller thermal conductivity than AgInTe_2_. The thermal conductivities for AgInTe_2_ and AgGaTe_2_ [16] yielded by experiments confirm what we had anticipated.

**Figure 4.**
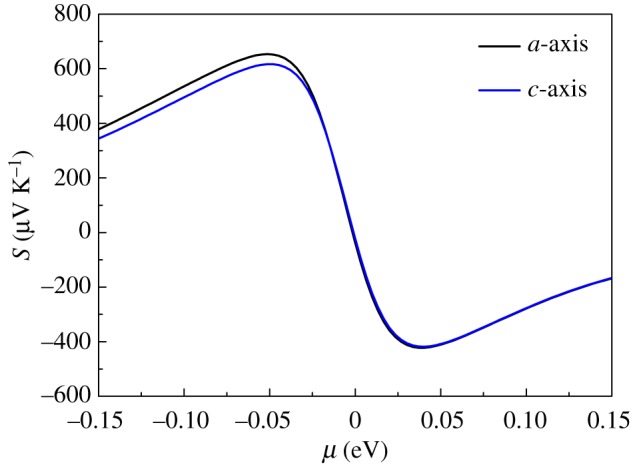
Calculated Seebeck coefficients as a function of chemical potential along the *a*- and *c*-directions for AgInTe_2_ at 300 K.

The Boltzmann transport theory in the form of CSTA can directly yield Seebeck coefficients. The Seebeck coefficients as a function of chemical potential for AgInTe_2_ at 300 K along the *a-* and *c*-directions are shown in figure 4. The Seebeck coefficients display anisotropy for the p-type AgInTe_2_ compound and show isotropy for the n-type one. Additionally, the Seebeck coefficient for the p-type AgInTe_2_ in the *a*-direction is larger than that in the *c*-direction, which mainly stems from the fact that the effective mass of holes in the *a*-direction is significantly larger than that in the *c*-direction as mentioned above. Furthermore, the Seebeck coefficient of the p-type AgInTe_2_ along the *c*-direction is larger than that of the n-type one, due to the fact that DOS near VBM increases sharply with an energy decrease as indicated in figure 2*a*.

In the form of CSTA, the electronic conduction *σ* cannot be independently calculated directly from the electronic structure by using the Boltzmann transport theory. In order to obtain *σ*, the information of the relaxation time *τ* is needed. We fit the theoretical total *σ*/*τ* with experimentally measured *σ* at a fixed temperature and carrier concentration to determine the behaviour of *τ*. We take advantage of the experimental data (*σ* = 2132.2 S m^−1^, *S* = 390 µV K^−1^) at 700 K [42] to fit the relaxation time. We use the average *S* and *σ*/*τ* along the *a-*, *b-* and *c*-directions to compare with experimental data, because the experimental data [42] are tested for arbitrary direction. The corresponding carrier concentration is 2.02 × 10^19^ cm^−3^ when the average *S* equals 390 µV K^−1^ at 700 K. Our calculated *σ*/*τ* equals 9.15 × 10^17^ S m^−1^ _S_^−1^ at the same carrier concentration. Comparing calculated *σ*/*τ* with experimental *σ*, we get the relaxation time at 700 K and 2.02 × 10^19^ cm^−3^ carrier concentration for AgGaTe_2_. The fitted relaxation time at 700 K and 2.02 × 10^19^ cm^−3^ carrier concentration for the AgGaTe_2_ compound is 2.33 × 10^−15^ s, which is a rational magnitude in semiconductors. Generally, *τ* decreases with an increase in temperature (*T*) and carrier concentration (*n*). We take the relaxation time *τ* in the form of *τ* = *AT*^−1^*n*^−1/3^. The fitted constant *A* at 700 K and 2.02 × 10^19^ cm^−3^ carrier concentration for the AgGaTe_2_ compound is *A* = 4.44 × 10^−6^. Thus, the relaxation time *τ* as an expression of *T* and *n* for the AgGaTe_2_ compound is presented as *τ* = 4.44 × 10^−6^*T*^−1^*n*^−1/3^. The relaxation time *τ* for the AgInTe_2_ compound is obtained using the same approach as that of AgGaTe_2_ because of their similar crystal structures.

**Figure 5.**
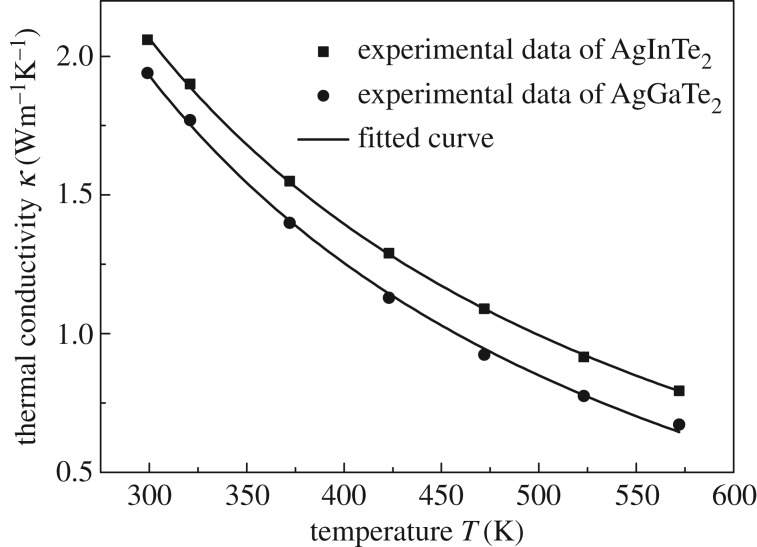
Fitted thermal conductivity curves for AgInTe_2_ and AgGaTe_2_ compounds.

We now discuss thermal conductivity. Thermal conductivity is contributed by both the electron and the lattice. The lattice thermal conductivity is the main contributor (over 98%) and is determined mostly by the lattice structure [43]. We assume the thermal conductivity only depends on temperature, which is widely used in thermoelectric materials [44,45]. Thermal conductivity is adopted by fitting the experimentally measured data at different temperatures: k=A+(B/T), where *A* and *B* are fitted constants. Figure 5 plots the temperature dependence of fitting thermal conductivity (solid lines) and the experimentally measured data [16] (scattered dots) for AgInTe_2_ and AgGaTe_2_ compounds. The thermal conductivities for AgInTe_2_ and AgGaTe_2_ compounds decrease greatly as the temperature increases. The temperature dependence of thermal conductivity for AgInTe_2_ and AgGaTe_2_ compounds can be obtained by using the formulae k=(801.86/T)−0.61 and k=(810.49/T)−0.77, respectively.

**Figure 6.**
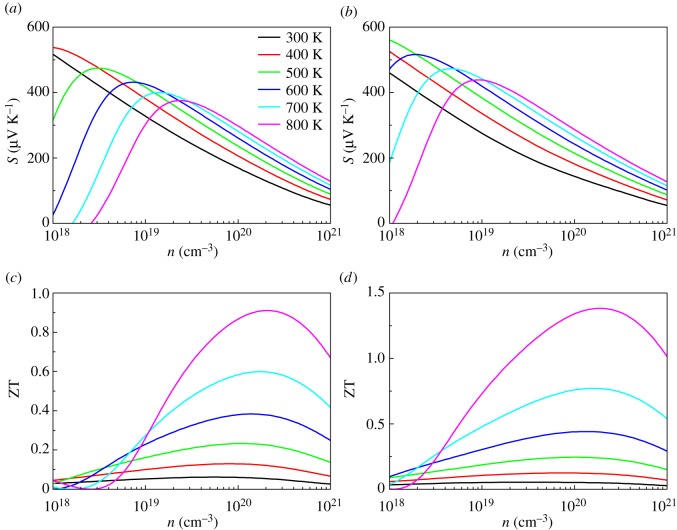
Calculated thermoelectric properties for AgX(In, Ga)Te_2_: (*a*) Seebeck coefficients for AgInTe_2_, (*b*) Seebeck coefficients for AgGaTe_2_, (*c*) thermoelectric figure of merit for AgInTe_2_ and (*d*) thermoelectric figure of merit for AgGaTe_2_.

The calculated Seebeck coefficients and ZT values for AgInTe_2_ and AgGaTe_2_ compounds at different temperatures with carrier concentration are given in figure 6. The optimized ZT values of AgIn(Ga)Te_2_ compounds at different temperature are listed in table 2. The Seebeck coefficients are sensitive to carrier concentration and temperature. The bipolar effects are obvious both for AgInTe_2_ and AgGaTe_2_ at high temperatures and low carrier concentrations, which result in the peaking of Seebeck coefficients at larger concentrations as the temperature rises from 300 to 800 K. There are two carrier concentrations, i.e. holes and electrons, in thermoelectric materials. Holes are mostly the carriers in p-type thermoelectric material while electrons prevail as carriers in the n-type one. For p-type thermoelectric material, the electronic concentration and electronic thermal excitation are obvious at low hole concentrations and high temperatures. We discover the bipolar effect of AgInTe_2_ is a little more evident than that of AgGaTe_2_. The reason is that the smaller band-gap of AgInTe_2_ makes electronic thermal excitation a little easier than that of AgGaTe_2_.

**Table 2.** Optimized ZT values of AgIn(Ga)Te_2_ compounds at different temperature. The corresponding carrier concentration, Seebeck coefficient, electronic conductivity and relaxation time are also listed.

	*T* (K)	*n* (cm^−3^)	*S* (µV K^−1^)	*σ* (10^3 ^S m^−1^)	*τ* (s)	ZT
AgInTe_2_	300	3.66 × 10^19^	206	10.00	3.88 × 10^−15^	0.06
	400	8.09 × 10^19^	221	9.28	2.57 × 10^−15^	0.13
	500	1.12 × 10^20^	228	8.90	1.85 × 10^−15^	0.23
	600	1.38 × 10^20^	236	8.31	1.43 × 10^−15^	0.38
	700	1.78 × 10^20^	237	8.16	1.13 × 10^−15^	0.60
	800	2.12 × 10^20^	240	7.78	9.32 × 10^−16^	0.91
AgGaTe_2_	300	2.83 × 10^19^	207	8.05	4.86 × 10^−15^	0.05
	400	7.01 × 10^19^	202	9.59	2.69 × 10^−15^	0.12
	500	1.09 × 10^20^	209	9.53	1.86 × 10^−15^	0.24
	600	1.29 × 10^20^	225	8.45	1.46 × 10^−15^	0.44
	700	1.54 × 10^20^	235	7.70	1.18 × 10^−15^	0.77
	800	1.97 × 10^20^	236	7.49	9.55 × 10^−16^	1.38

As can be seen, the figure of merit ZT heavily depends on carrier concentration and temperature. The bipolar effects are clearly visible, which cause the ZT at 800 K to be lower than the ZT at 700 K at low carrier concentrations. The maximum ZT values for AgInTe_2_ and AgGaTe_2_ at 800 K are 0.91 and 1.38 at approximately 2.12 × 10^20^ cm^−3^ and 1.97 × 10^20^ cm^−3^ carrier concentrations, respectively. This indicates AgGaTe_2_ is a potential high-temperature thermoelectric material for use in thermoelectric devices.

## 4. Conclusion

We have presented the electronic, vibrational and thermoelectric transport properties of AgInTe_2_ and AgGaTe_2_ in the form of the first-principles theory. The electronic structures are obtained using the PBE + U approach and the results agree well with existing experimental results. The calculated effective mass shows that AgInTe_2_ and AgGaTe_2_ are possible p-type thermoelectric materials and thermoelectric properties are anisotropic in different crystallographic directions. The results of vibrational transport properties calculated using harmonic approximation anticipate the low thermal conductivity of AgInTe_2_ and AgGaTe_2_. Thermoelectric properties including Seebeck coefficients and figures of merit for AgInTe_2_ and AgGaTe_2_ are obtained using the Boltzmann transport theory in the form of CSTA based on first-principles electronic structures and previous experimental results. The bipolar effects at low doping levels and high temperatures for AgInTe_2_ and AgGaTe_2_ are clearly visible and have a significant influence on the thermoelectric properties. When we compare the thermoelectric properties between AgInTe_2_ and AgGaTe_2_, the ZT value of AgGaTe_2_ at 800** **K is 1.38 at a doping level of about 1.97 × 10^20^ cm^−3^, which confirms AgGaTe_2_ is a potential thermoelectric material at a high temperature.

## Data accessibility

The data have been deposited at the Dryad Digital Repository (http://dx.doi.org/10.5061/dryad.t10p2) [46].
